# Neural mechanisms of attentional control in mindfulness meditation

**DOI:** 10.3389/fnins.2013.00008

**Published:** 2013-02-04

**Authors:** Peter Malinowski

**Affiliations:** School of Natural Sciences and Psychology, Liverpool John Moores UniversityLiverpool, UK

**Keywords:** meditation, mindfulness, attentional control, Stroop, attention

## Abstract

The scientific interest in meditation and mindfulness practice has recently seen an unprecedented surge. After an initial phase of presenting beneficial effects of mindfulness practice in various domains, research is now seeking to unravel the underlying psychological and neurophysiological mechanisms. Advances in understanding these processes are required for improving and fine-tuning mindfulness-based interventions that target specific conditions such as eating disorders or attention deficit hyperactivity disorders. This review presents a theoretical framework that emphasizes the central role of attentional control mechanisms in the development of mindfulness skills. It discusses the phenomenological level of experience during meditation, the different attentional functions that are involved, and relates these to the brain networks that subserve these functions. On the basis of currently available empirical evidence specific processes as to how attention exerts its positive influence are considered and it is concluded that meditation practice appears to positively impact attentional functions by improving resource allocation processes. As a result, attentional resources are allocated more fully during early processing phases which subsequently enhance further processing. Neural changes resulting from a pure form of mindfulness practice that is central to most mindfulness programs are considered from the perspective that they constitute a useful reference point for future research. Furthermore, possible interrelations between the improvement of attentional control and emotion regulation skills are discussed.

## Introduction

Recent years have seen a burgeoning interest in mindfulness-based approaches, primarily driven by growing evidence of their beneficial effects on physical and mental well-being. In parallel to research evaluating the effectiveness of these approaches, a second line of investigation concentrates on unraveling the psychological and neurophysiological processes involved. A more precise understanding of these processes will facilitate the refinement of mindfulness-based interventions and will allow the development and fine-tuning of programs that account for specific psychological or physiological conditions and cater for individual circumstances and predispositions. Several theoretical propositions have already been made. For example, neurobiological processes of wanting and liking may be of great importance when supporting people with addictions or binge eating disorders (Kristeller and Wolever, 2011), while the monitoring and self-regulation of cognitive and emotional states may be emphasized in programs tailored to the needs of individuals with Attention Deficit Hyperactivity Disorder (Zylowska et al., 2008). Programs addressing recurrent depression may focus on recognizing and stepping out of automatic modes of thinking and feeling (Kuyken et al., 2008) and the development of self-determination and resilience has been suggested for the treatment of severe mental illness (Davis and Kurzban, 2012).

To consolidate these largely theoretical propositions, it will be crucial to advance our understanding of the underlying cognitive, emotional, and neural processes. The refinement of attention regulation skills features centrally in all conceptualizations of mindfulness training and recent neurophysiological evidence shows that regular, brief engagement in a simple mindfulness meditation significantly improves attentional control processes (Moore et al., 2012). These results provide important insights into the development of core processes of mindfulness and establish a useful reference point when investigating the effects of more elaborate or expanded practices, or when considering the interactions between attention and emotion regulation skills.

### Mindfulness

The majority of psychological and neuroscientific studies into **mindfulness** adopt a definition put forward by Jon Kabat-Zinn, who was pivotal in translating Buddhist approaches of mind training into the secular context of health care programs and psychological interventions (e.g., Kabat-Zinn et al., 1985, 1992; Kabat-Zinn, 2011). He describes mindfulness as “the awareness that emerges through paying attention on purpose, in the present moment, and non-judgmentally to the unfolding of experience moment by moment”(Kabat-Zinn, 2003). This general understanding is echoed by other authors who explain mindfulness as being “characterized by dispassionate, non-evaluative, and sustained moment-to-moment awareness of perceptible mental states and processes. This includes continuous, immediate awareness of physical sensations, perceptions, affective states, thoughts, and imagery” (Grossman et al., 2004) or as “a receptive attention to and awareness of present events and experience” (Brown et al., 2007).

While significant differences exist between Buddhist views of mindfulness and modern psychological adaptations, there is broad agreement that a clearly formulated mental training, usually referred to as **meditation**, is required for developing and improving levels of mindfulness (Chiesa and Malinowski, 2011).

### The liverpool mindfulness model

**Figure 1:**
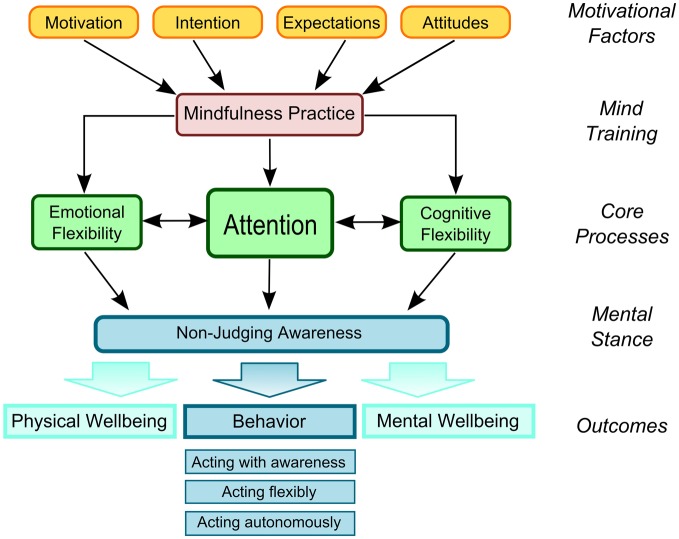
**The Liverpool Mindfulness Model**.

The Liverpool Mindfulness Model presented in Figure 1 aims to capture and integrate the core components that are involved in mindfulness practice and to provide a framework for directing future research (Malinowski, 2012). Consistent with other conceptualizations of mindfulness meditation practice, the model gives the development of attentional skills a central role in this process (Wallace and Shapiro, 2006; Lutz et al., 2008; Tang and Posner, 2009; Hölzel et al., 2011; Slagter et al., 2011).

The model structures the process into five main tiers: the driving *motivational factors* (tier 1) determine whether and how an individual engages in the *mind training* (tier 2). Regular engagement in mindfulness practice develops and refines the mental *core processes* (tier 3), primarily based on the refinement of attentional functions that interact with and facilitate regulatory processes of emotions and cognitions. Improvements in these core processes result in a changed and more balanced *mental stance* or attitude (tier 4), that will result in a positive *outcome* (tier 5) in terms of physical and mental well-being, and the quality of behavior. Each tier and component of this model, as well as the interactions and assumed causal relationships between them, warrant further detailed research and render the model a suitable roadmap in this endeavor.

### Mindfulness, meditation, and attention

Training and refining attention skills are central to most psychological and Buddhist conceptualizations of mindfulness practices (Lutz et al., 2008) and are the main concern of this review. As outlined in Figure 1, the training of attention skills is thought to underpin emotional and cognitive flexibility, bringing about the ability to maintain non-judging awareness of one's own thoughts, feelings, and experiences in more general terms. This, in turn, will change the quality of one's behavior and lead to positive health outcomes and well-being (Wallace and Shapiro, 2006; Chiesa and Malinowski, 2011; Malinowski, 2013). Meditations that calm and stabilize the mind are of central importance in this process and are prerequisite for a second, more advanced class of meditations (Wallace, 1999; Lutz et al., 2008; Malinowski, 2008, 2013). These two forms of training have been explained as **Focused Attention** (FA) and **Open Monitoring** (OM) meditation practices (Lutz et al., 2008), respectively. Although conceptually FA and OM can be separated, even simple forms of mindfulness training will entail both components. Initially a practitioner will engage more with the FA component to develop attentional stability, clarity, and awareness of the current mental state. Only then will it be possible to engage in a meaningful way in OM practice, which entails a moment by moment attentiveness to anything that occurs in experience. With increasing experience, OM practice will become less reliant on FA and can eventually be maintained without focusing on any explicit object. These fundamental principles are captured by common psychological definitions of mindfulness that emphasize the development of attentional abilities combined with a specific, non-evaluative attitude toward the different mental experiences that may arise (e.g., Bishop et al., 2004; Shapiro et al., 2006; Malinowski, 2008, 2013; Chiesa and Malinowski, 2011).

**Figure 2:**
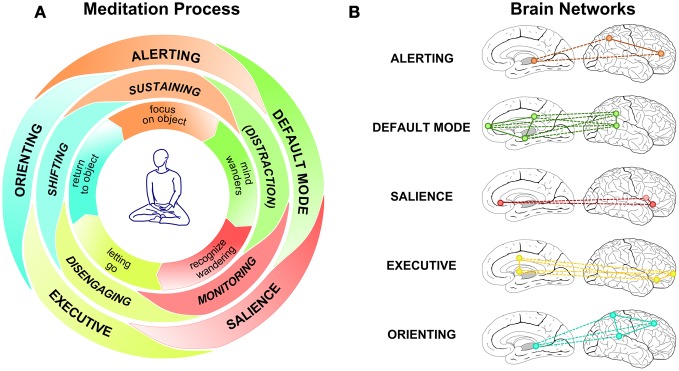
**Effortful attention regulation during meditation.** Panel **(A)** provides a schematic representation of the meditation process. The inner circle outlines the phenomenological layer, presenting the typical sequence (clockwise) a meditator will go through. The middle circle relates the attentional processes that lie underneath, while the outer circle represents the different brain networks that are involved in carrying out these functions. The different attentional processes and the brain networks are represented as partially overlapping to indicate that in many instances more than one process/network is involved. Panel **(B)** outlines the main brain areas involved in each of the five networks. Anatomical details are discussed in the main text.

Of particular interest to this review are the attentional processes that constitute the backbone of these practices. Within cognitive neuroscience attention is commonly thought of in terms of three main functions: (1) the modulation of arousal, alertness, and attentional engagement, (2) the function of stimulus selection, and (3) the function of attentional control processes. Three different, though interrelated, **attentional networks** subserve these functions, the *alerting, orienting*, and *executive control* networks, respectively (Posner and Petersen, 1990; Corbetta and Shulman, 2002; Fan et al., 2005; Raz and Buhle, 2006; Posner and Rothbart, 2007). Figure 2B provides a schematic presentation of the brain areas associated with these networks. The right frontal and right parietal cortex and the thalamus are involved in alerting functions. The superior parietal cortex, temporal parietal junction, frontal eye fields, and superior colliculus are involved in orienting. The anterior cingulate cortex (ACC), lateral ventral cortex, prefrontal cortex, and basal ganglia contribute to executive control processes (Fan et al., 2005; Posner and Rothbart, 2007). Recent neuroimaging evidence further subdivides the function of the latter network, suggesting that the dorsal ACC, the ventrolateral prefrontal cortex, and the neighboring anterior insula constitute a *salience network*. This network is involved in the attentional control function of detecting subjectively relevant or salient events across modalities (cognitive, homeostatic, or emotional) and provides signals to the executive network to act upon in accordance with the current goal set (Dosenbach et al., 2006, 2007; Seeley et al., 2007; Sridharan et al., 2008). Finally, whenever attention involuntarily drifts away from the object during meditation, a further network will become involved, the *default mode network*, which entails the posterior cingulate cortex, the medial prefrontal cortex, the posterior lateral parietal/temporal cortices, and the parahippocampal gyrus (Mason et al., 2007; Buckner et al., 2008; Hasenkamp et al., 2012). This network has been shown to be activated as soon as participants involuntarily engage in task-unrelated cognitions or mind wandering (e.g., Mason et al., 2007; Buckner et al., 2008; Schooler et al., 2011). Tang et al. (2012) recently presented a similar view on this topic.

Figure 2A summarizes the assumed process of focused meditation by considering three layers: the phenomenological experience of the meditator, the underlying attentional processes, and the brain networks subserving these processes. On the phenomenological level the meditator will engage with the practice by focusing on the relevant meditation object, for instance, the somatosensory sensation accompanying ones breathing. During this phase of sustaining attention, the alerting network will be involved. In the moment the mind loses the focus on the object and mind wandering occurs, the default mode network will become more active. Sooner or later the meditator will recognize the mind wandering by means of the attention monitoring function and the involvement of the salience network. When mind wandering is detected, the meditator lets go of the distracting train of thought or experience by means of attentional disengagement and the involvement of the executive network. The subsequent return to the meditation object is achieved by shifting the focus back to the object, a function of attention involving the executive and the orienting network. This process can unfold within a few brief moments or can extend over longer periods of time. With increasing levels of expertise, periods of sustained focus and attentional stability may become more and more extensive (Wallace, 2006), whereas for a beginner, even longer periods of mind wandering may pass unnoticed. Although described as separate, these processes and brain network activations may indeed overlap and occur in parallel, expressed in Figure 2A by rendering the components of the middle and outer circle as partially overlapping. For instance, evidence from **functional Magnetic Resonance Imaging** (fMRI) indicates sustained activity in the salience network during meditation (Baron Short et al., 2010). Furthermore, and in line with the process model presented here, Hasenkamp et al. (2012) used fMRI to study brain network activity when meditators shifted between periods of mind wandering and of sustained focus, concluding that the salience network signals the detection of mind wandering to the executive network. This, in turn, would initiate a re-orienting of attention to the object of meditation.

## Converging evidence: the role of attention

Evidence gained with a variety of methodological approaches clearly indicates that mindfulness meditation increases the efficiency of attentional functions, reflected in performance increases as well as changes in neural activity and underlying neural architecture.

### Sustained attention

Robertson et al. (1997) defined sustained attention as “the ability to self-sustain mindful, conscious processing of stimuli whose repetitive, non-arousing qualities would otherwise lead to habituation and distraction to other stimuli” (p. 747). This definition captures the central features of mindfulness practice and indicates the relevance of sustained attention in this process. Studying a student sample without significant meditation experience, Schmertz et al. (2008) found that higher self-reported mindfulness was related to measures of more stable attention in the Continuous Performance Test (Conners, 2000), a test frequently used for assessing sustained attention. Similarly, Moore and Malinowski (2009) reported a positive correlation between self-reported mindfulness and performance on the d2-test of attention (Brickenkamp and Zilmer, 1998). Furthermore, mindfulness practitioners performed significantly better on this test than their matched non-meditating controls (Moore and Malinowski, 2009). Similarly, Valentine and Sweet (1999) and Pagnoni and Cekic (2007) reported better performance of meditators in sustained attention tasks. In other studies the attentional blink paradigm was employed to investigate how a three-month intensive meditation retreat improves meditators ability to sustain the focus of attention, as compared to a non-meditating matched control group (Slagter et al., 2007, 2009). The attentional blink task requires participants to attend to a rapidly changing stream of stimuli (e.g., letters) and to report the identity of two embedded target stimuli (e.g., digits) after each trial. Performance to the second target in the stream typically suffers if it appears within 500 ms after the first target, the so-called attentional blink effect (Shapiro et al., 1997). This performance detriment was significantly reduced after the meditators had completed their meditation retreat. In parallel, the amplitude of the P3b **event-related potential** (ERP) elicited by the first target stimulus, was decreased in meditators. The participants with the greatest decrease of the P3b amplitude also showed the largest decrease in attentional blink size (Slagter et al., 2007). Because the P3b component is considered to index the allocation of **attentional resources**, these results suggest that the meditation training improved the meditators ability to sustain attentional engagement in a more balanced and continuous fashion. This was expressed as enhanced allocation of neural resources (Wickens et al., 1983; Marois and Ivanoff, 2005), which facilitated the detection of the second target. An additional analysis of the phase of oscillatory theta activity following successfully detected second targets showed a reduced variability across trials, a signature of more consistent deployment of attention in meditators (Slagter et al., 2009). Taken together these findings indicate improved efficiency in engaging and disengaging from relevant target stimuli (Lutz et al., 2008), i.e., flexibility of allocating attentional resources.

### Attentional control

**Figure 3:**
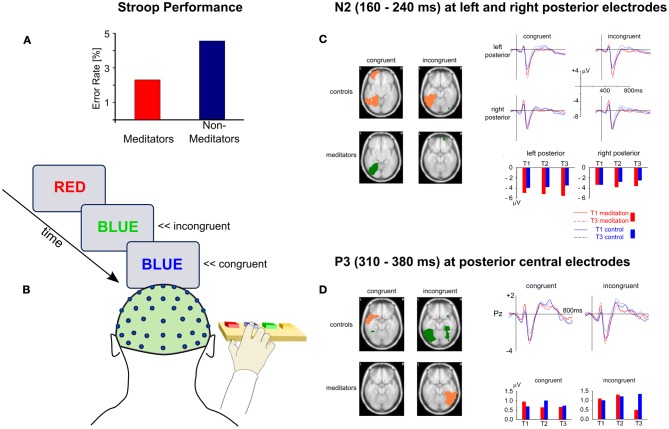
**Behavioral and ERP results from the Stroop task as a function of meditation practice. (A)** Performance differences between meditators and non-meditators in a cross-sectional comparison (Moore and Malinowski, 2009). **(B)** Outline of the Stroop task. **(C)** and **(D)** Results from a longitudinal study (Moore et al., 2012) showing effects of meditation training on ERPs during the Stroop task for the N2 **(C)** and P3 **(D)** ERP components. The glass brain slices show activation differences between T1 and T3 for each group and congruency. Salmon-colored areas indicate a decrease in activation and green areas indicate activation increase. On the right hand side ERPs (line graphs) and ERP-component amplitudes (bar graphs) are depicted for left and right posterior sites **(C)** and for posterior central sites **(D)**.

As Figure 2 schematically outlines, sustaining focused attention over extended periods of time requires the interplay of several attentional processes. Of particular importance is the ability to monitor and regulate ones attentional state or—during task performance—one's responses. The majority of the employed paradigms discussed so far were not geared toward separating out the involvement of the different attentional functions or networks. Importantly, most tasks tapping sustained attention will also recruit attentional control functions, such as the monitoring and updating of information, mental set shifting, and the inhibition of proponent, but non-relevant responses (Miyake et al., 2000). Similarly, because the mental practice of meditation requires the monitoring and adjustment of one's attentional focus, control processes will be crucially involved, at least until a level of expertise is achieved where attentional stability can be maintained with little or no effort, possibly well beyond 19,000 h of accumulated meditation practice (Brefczynski-Lewis et al., 2007; Tang et al., 2012). Given the central role of these monitoring and control processes in developing such stability, several studies into attentional functions and meditation focus on these processes, frequently by employing the Stroop Word-Color Task (Stroop, 1935)—a canonical measure of response inhibition (Macleod, 1991; Miyake et al., 2000). The task requires participants to rapidly name or indicate the color of the font a word is presented in (see Figure 3B). The highly automatized function of reading leads to performance decrements (slower responses and/or higher error rates) in the incongruent condition, i.e., when the meaning of a color word conflicts with its font color (e.g., “GREEN” presented in red). High proficiency in this task is thus thought to indicate good attentional control and relatively low automaticity or impulsivity of one's responses.

Employing cross-sectional comparisons, several studies reported significantly better performance for meditators than non-meditators on this task (see Figure 3A) and found that task performance was also related to lifetime meditation experience (Chan and Woollacott, 2007; Teper and Inzlicht, 2013) and levels of self-reported mindfulness (Moore and Malinowski, 2009). Similarly, compared to an active control condition, significant improvements in Stroop performance were observed when mindfulness was induced by means of three 20-minute mindfulness sessions, delivered via audio-recording (Wenk-Sormaz, 2005). However, a study by Anderson et al. (2007) failed to find improvements in Stroop performance after an 8-week mindfulness-based stress reduction (MBSR, Kabat-Zinn, 2003) program. Because Anderson et al. used an atypical Stroop task and the MBSR program consists of a broad range of components, some of which are not directly related to meditation, drawing general conclusions is difficult. Nevertheless the study highlights the need for purer designs that do not conflate too many components. Employing the Attention Network Test (ANT, Fan et al., 2002) Jha et al. (2007) reported better executive control performance of meditators compared to controls in line with Tang et al. (2007) who found meditation-specific improvements in this measure after five 20-minute sessions of mindfulness training.

However, to gain an understanding of the cognitive and neurophysiological processes that are reflected in performance changes on attentional control tasks, it is important to study the underlying mechanisms in a purer and more detailed fashion. By conducting an extensive longitudinal, wait-list controlled study, Moore et al. (2012) contributed to this in important ways. Participants new to meditation practice engaged in daily 10-minute sessions of mindful breathing meditation over a period of 16 weeks and performed the Stroop task before (T1), half-way through (T2), and after completion of the 16-week meditation period (T3). ERPs were recorded concurrently to study the neuronal changes of attentional control processes. The results showed that meditation practice influenced the neuronal responses to the Stroop stimuli in two important ways. Firstly, it led to a relative increase of lateral posterior N2 amplitudes (160–240 ms) over both hemispheres, irrespective of stimulus congruency (Figure 3C). These changes in the meditation group were primarily driven by increased activity in the left medial and lateral occipitotemporal areas for congruent stimuli, which was contrasted by decreased activity in similar brain areas in the control group. The second difference between meditators and controls was observed in the P3 component, peaking between 310 and 380 ms, primarily for incongruent stimuli. While the participants in the control group exhibited an increase of the P3 amplitude for incongruent stimuli, a decrease was observed for the meditation group, attributed to reduced activity in lateral occipitotemporal and inferior temporal regions of the right hemisphere (Figure 3D).

However, a third finding was at odds with what the majority of studies found before. Improvements in Stroop performance from T1 to T3 were as large in the control group as they were in the meditators. Reflecting these behavioral outcomes, meditators and controls did not differ regarding a typical neural signature of response conflict, a negative ERP deflection peaking between 400 and 600 ms post stimulus, which is usually correlated with task performance (Liotti et al., 2000). Thus, although clear evidence for better Stroop performance of meditators than non-meditators has been found in cross-sectional comparisons, it did not emerge in the same way in a longitudinal study. A possible explanation might be that the repeated administration of the same task mandated by the longitudinal design lead to a performance ceiling. This suggestion is supported by the fact that performance did not improve in either group after T2 and that accuracy was above 95% for incongruent trials.

An alternative and possibly related explanation concerns the involvement of the ACC. The ACC is known to be the generator of the late negative ERP (Liotti et al., 2000; Hanslmayr et al., 2008) that usually correlates with Stroop performance but was not differentially influenced by meditation experience. fMRI evidence suggests that the ACC is more involved in the anticipatory regulation of attention rather than the specific selection of responses itself (Roelofs et al., 2006; Aarts et al., 2008). It is conceivable that with extended task exposure this anticipatory regulation was perfected in both groups, possibly resulting in the observed ceiling effect.

A recently published study offers a further explanation for the lack of meditation-specific behavioral effects. Teper and Inzlicht (2013) investigated attentional control mechanisms in the Stroop task by focusing on the neural processes involved during the response phase, rather than on the stimulus processing stage discussed so far. The error-related negativity (ERN), a neurophysiological response that occurs within 100 ms after participants commit an incorrect response, is considered to be a useful marker of performance monitoring processes (Falkenstein et al., 2000; Yeung et al., 2004) and has also been linked to affect and motivation (Ganushchak and Schiller, 2008; Weinberg et al., 2012). The authors found enhanced ERN amplitudes in meditators compared to controls after a Stroop error was committed. Further analysis revealed that meditation experience improved attentional control primarily in an indirect way, by fostering the acceptance of emotional states, an aspect of mindful emotion regulation abilities that was assessed by self-report (Philadelphia Mindfulness Scale, Cardaciotto et al., 2008). In line with this, comparing participants who scored high vs. low on emotional acceptance a trend toward enhanced ERN amplitudes for high emotional acceptance was found (Teper and Inzlicht, 2013). The finding that performance on executive control tasks was affected by emotion regulation abilities might explain why cross-sectional studies tend to find performance differences, whereas the longitudinal study did not. It seems plausible that emotional influences are more prevalent during the first contact with the task, whereas the emotional component “wears off” following repeated exposure within a longitudinal design and related performance differences decrease.

If this interpretation holds true, better Stroop performance in meditators, commonly attributed to de-automatization, may—at least partially—be due to less emotional reactivity and may thus reflect improved emotion regulation strategies rather than attentional control processes. This perspective highlights the close link between attention regulation and emotion regulation skills (also see Figure 1) and raises a question concerning their refinement: do improvements in emotional regulation skills precede those in cognitive processing or vice versa and are executive control processes the basis for improved emotion regulation skills? The latter relationship is certainly what phenomenological accounts of mindfulness practice would suggest (e.g., Wallace and Shapiro, 2006; Lutz et al., 2008) and is in line with evidence from two recent studies. Sahdra et al. (2011) reported that participation in a three-month intensive meditation retreat concurrently resulted in enhanced response inhibition performance and improved socio-emotional functioning as measured by a broadly conceived composite measure of adaptive socio-emotional functioning (consisting of 14 self-report measures such as emotion regulation, depression, anxiety, well-being, ego resilience, empathy, etc.). Further analysis revealed that the socio-emotional functioning was influenced by enhancement of response inhibition skills, lending support to the hypothesis that attentional control skills may underpin the development of emotion regulation skills. Allen et al. (2012) used fMRI to investigate neural changes in cognitive and emotional processing resulting from six weeks of meditation training. Using an emotional Stroop task which included the presentation of affective stimuli with positive or negative valence, the study found that the conflict scores only diminished in the meditation group but not in the active control group. This was accompanied by a meditation-related increase in activation of the dorsolateral prefrontal cortex during the task. As this area is involved in the executive control network (Raz and Buhle, 2006; Seeley et al., 2007; also see Figure 2) this finding may be interpreted as an improvement in attentional control. Interestingly, the total time participants had invested in the meditation practice was positively related to increased activity in areas implicated in the salience network, such as the anterior insula and the cingulate cortex (Seeley et al., 2007; Buckner et al., 2008; also see Figure 2). These findings are in line with the hypothesized progression from improvements of attentional control, indexed by the involvement of the executive control network, to improved emotion regulation skills, indexed by the selective involvement of the salience network. However, it should be noted that the participants in that study progressively engaged in four mindfulness practices (from focused breath awareness, to body-scanning, to compassion and to open monitoring) that progressively require increasing emotional awareness. Presumably, the most dedicated participants will also have engaged more with those emotional awareness practices and would thus exhibit more emotion related changes. Thus, while the data of these studies are in line with the assumption that with growing expertise the meditator progresses from attention regulation to emotion regulation, the results are not yet conclusive and studies that focus specifically on this question are required.

Against the backdrop of these studies, the main findings by Moore et al. (2012) are of high significance as they clearly outline the specific neural processes related to attentional control processes that result from one simple form of mindfulness practice. The enhancement of the N2 component and the associated increase of activity in left-hemispheric areas of the ventral processing stream (medial and lateral occipitotemporal areas) likely reflect more successful or consistent attentional amplification, specific to the features of the color words used in the task, contrasting with decreased activation due to habituation in the control group. This interpretation seems plausible as these brain areas are typically involved in lexical tasks (Cohen et al., 2002; Cohen and Dehaene, 2004; Shaywitz et al., 2004) with a similar posterior N2 component (Adorni and Proverbio, 2009) and the time course fits to the observed attentional enhancement of color as compared to form stimuli (Eimer, 1997). The evidence thus shows that engaging in a simple mindful-breathing practice improves the ability to selectively allocate attentional resources to task-relevant features—in this case the color of a lexical stimulus.

The reduction of the P3 component during the processing of incongruent color words, attributed to the decrease of activity in lateral occipitotemporal and inferior temporal regions of the right hemisphere, appears to reflect more efficient attentional resource allocation during perceptual stimulus discrimination and inhibition processes that are required for resolving the conflicting stimulus information (Polich, 2007). A recent fMRI study comparing meditators and matched controls on the Stroop task reports reduced activity in various brain areas subserving attention (Kozasa et al., 2012), lending further support to the idea of enhanced neuronal efficiency resulting from meditation practice. But more precisely, Moore et al. (2012) show that this more efficient resource allocation relates to those perceptual discrimination processes that require a higher degree of attentional control.

Before concluding that the observed effects are specific to the meditation practice it is worth considering the suggestion that the observed changes to stimulus processing occurred as a by-product of the meditation process. As meditators tend to practice either with closed or half-open eyes the resulting reduction of sensory load may have increased the excitability of the visual cortex, a phenomenon that has been observed also after shorter periods of sensory deprivation (Suedfeld, 1975; Boroojerdi et al., 2000; Pitskel et al., 2007). In turn, the reduced threshold for sensory stimuli may have enhanced the related ERP components. While such an effect of meditation practice needs to be considered it seems unlikely that it plays an important role here. The fact that the laboratory-based Stroop task was completed independently of meditation practice, that the participants only practiced for about 10 min/day, and that no differences between meditators and controls were observed in the early visual ERP components P1 or N1, speak against such interpretation. Nevertheless, it will be useful to consider (and control) such influences in future research, in particular when studying the influence of more prolonged meditation regimes, or when recording the ERPs soon after (or while) participants engaged in formal practice.

## Conclusion

Longitudinal studies indicate that meditation practice results in significant changes to earlier stimulus processing in terms of enhanced/more consistent, dynamic, and flexible attentional functions. Improvements in attentional selection and control appear to be primarily mediated by more flexible attentional resource allocation that modulates early stimulus processing, possibly in a modality independent fashion. Rather than enhancing response inhibition processes per se, the study by Moore et al. (2012) revealed meditation-related improvements to earlier stages of stimulus processing in terms of more focused attentional resources (indexed by the enhanced N2) and more efficient perceptual discrimination and conflict resolution processes (indexed by the reduced P3). When considering these two findings together, an interesting interpretation emerges: the more successful attentional amplification of the color word stimuli may have influenced the subsequent object recognition processes in positive ways, so that less attentional resources needed to be invested.

Specific conclusions can be drawn because the study was confined to one specific, simple meditation practice, rather than the more complex or varied forms of meditation that were the focus of the majority of previous longitudinal studies into meditation. It seems that mindfully focusing on the somatosensory experiences of breathing leads to specific improvement to core processes of attentional control that are considered to be central to all forms of mindfulness practice. As this form of practice is the starting point for the majority of mindfulness meditation programs the findings are an important reference point for future research that aims to investigate more complex, advanced, or prolonged and extended mindfulness programs.

The reported improvements seem to generalize from the specific situation of a meditation exercise (i.e., focusing on breathing related sensations and maintaining a non-responsive attitude to all arising experiences) to a different sensory modality, in this case vision. Thus, a growing body of evidence provides strong support for the idea that improvements in attentional core processes of selection and control, and their related beneficial effects, may propagate into modalities different from the meditation practice itself, exerting positive effects in various situations, and for various conditions.

Although evidence regarding the role of attention is mounting, these are still early days and there are certainly more questions unanswered than answered. Due to a paucity of well controlled longitudinal studies, much of the available evidence is gained from cross-sectional comparisons, which are of limited use in unraveling the causal contribution of mind training to improvements in attentional functions. Therefore, more longitudinal studies that focus on specific psychological and neuronal mechanisms are required. Furthermore, such studies will need to consider the interplay of emotional and attentional factors in more detail to determine whether emotional flexibility improves attentional functions or vice versa.

The fact that a simple form of mind training exerts a clear influence on modality-independent attentional processes may indicate why mindfulness-based interventions prove to be beneficial in various situations. It appears that by refining the process of relating to experiences, rather than engaging with the content of experience, generic skills that can be applied across domains and modalities are enhanced.

### Conflict of interest statement

The author declares that the research was conducted in the absence of any commercial or financial relationships that could be construed as a potential conflict of interest.

## Key Concept

Mindfulness: Within the western psychological context, mindfulness is usually described as non-judgmental awareness of the present moment and is thought to entail paying attention with a certain attitude. It is commonly assumed that levels of mindfulness can be increased through meditation practice.
Meditation: Here describes mental practices carried out repeatedly to achieve specific positive outcomes. Mindfulness training and/or Buddhist practices usually entail aspects of calming and stabilizing the mind by training focused attention and of gaining a refined understanding of one's mental states by cultivating a non-elaborating, open, and observing attitude toward all arising mental events.
Focused attention: A form of meditation that involves the practice of sustaining the attentional focus on a chosen object, such as the sensation of ones breathing, and to return to the object as soon as mind wandering is detected. Importantly, the meditation object only serves as a neutral anchor or reference point which is not contemplated or evaluated during the process.
Open monitoring: This second form of meditation practice is firmly rooted in Buddhist forms of mind training. Building on attentional stability and clarity achieved with focused attention meditation, the aim here is to maintain an open, curious non-discriminating awareness of all arising sensations and mental events.
Attentional networks: Neuroscientific studies suggest that specific attentional functions are carried out by several interconnected brain networks. The attentional functions and related networks go under different names, but a classification into the three networks of alerting, orienting, and executive control is common. It is now understood that under many circumstances these networks interact and influence each other.
functional Magnetic Resonance Imaging: This method highlights differences in brain activity by measuring related blood oxygenation levels. It yields information regarding relative differences in brain activity when comparing two or more experimental conditions and thus offers useful insight as to which brain areas are selectively active during certain mental processes.
Event-related potential: The electrophysiological neural response measured on the scalp and directly related to a specific sensory, cognitive, or motor event. Event-related potentials (ERPs) are analyzed after the time-locked averaging of the neural response to several repetitions of the same event. Their positive and negative voltage deflections give an indication of specific neural processes related to the analyzed event.
Attentional resources: Neuronal processing capabilities which can be allocated to specific cognitive processes. Attentional resource theories often include capacity limits, although these do not need to be completely fixed.
